# Environmental Factors Modulate the Electronic Transitions and Molecular Vibrations of Lycopene: A Spectroscopy Perspective

**DOI:** 10.3390/molecules31132358

**Published:** 2026-07-03

**Authors:** Lu Xing, Shuping Zhao, Yeqiu Li, Yi Shi, Qin Dai, Wei Zhang

**Affiliations:** 1 School of Science, Shenyang Ligong University, No. 6 Nanping Central Road, Shenyang 110159, China; 2 School of Chemistry and Chemical Engineering, Nanjing University of Science and Technology, Nanjing 210094, China

**Keywords:** lycopene, Raman spectra, molecular orbital, first principle calculation

## Abstract

Lycopene is a highly significant carotenoid in daily life, exhibiting potent antioxidant properties and recognized as one of the most powerful natural antioxidants identified in plants to date. Its functionality originates from electronic and vibrational states that exhibit a high sensitivity to environmental perturbations. Nevertheless, exclusively experimental methodologies face challenges in delivering a comprehensive molecular-level comprehension of the influence exerted by particular environmental factors on the vibronic characteristics. This deficiency in understanding hinders the accurate prediction of its behavior and functional performance within complex systems. The first principle computational investigation enables a precise elucidation of the coupling mechanisms between electronic excitations and vibrational modes under diverse solvation and interaction environments. The results indicate that the local environment significantly influences the charge distribution and orbital energies of lycopene, altering its vibrational and electronic state properties. This provides a fundamental theoretical framework for predicting their photophysical behavior and biological functions within complex matrices.

## 1. Introduction

Lycopene is a natural carotenoid with the molecular formula C_40_H_56_ and a molecular weight of 536.85. In its pure form, it appears as deep red needle-like crystals [1]. Structurally, lycopene is an acyclic, planar, conjugated polyunsaturated hydrocarbon, characterized by the presence of 11 conjugated double bonds and 2 non-conjugated double bonds. It lacks terminal aromatic rings, exhibiting an open-chain configuration [2]. The structure of lycopene lacks the β-ionone ring, which prevents its enzymatic conversion into vitamin A precursors. Although lycopene and β-carotene share a similar isoprenoid backbone, the absence of the β-ionone ring in lycopene results in significant differences in their biological activities [3]. As a vital nutrient that cannot be endogenously synthesized by the human body, lycopene is classified as a natural carotenoid predominantly found in tomatoes and other fruits and vegetables. It exhibits significant physiological functions, particularly in antioxidation and disease prevention, including cancer, thereby holding substantial importance for human health [4]. Lycopene has been shown to activate both humoral and cellular immune responses, protecting phagocytic cells from oxidative damage, inhibiting the production of pro-inflammatory cytokines, and promoting immune homeostasis. These effects underscore its significant role in preventing inflammation, maintaining health, and reducing disease risk [5]. However, due to its lipophilic nature, lycopene exhibits low solubility in aqueous environments, which complicates its extraction and results in poor bioavailability. To address these limitations, recent studies have developed lycopene delivery systems, such as lipid-based nanoparticles, which markedly enhance its stability and water solubility, thereby improving its bioavailability [6].

To date, researchers have undertaken comprehensive investigations into the biological functions of lycopene, but bridging the gap between its macroscopic physiological effects and its molecular-level behavior under realistic environmental conditions remains a significant challenge [7,8,9]. The functional efficacy of this compound is intrinsically linked to its electronic and vibrational states, which are highly sensitive to external factors such as solvation and molecular interactions [10,11]. Experimental techniques often face limitations in isolating and quantifying the specific influence of individual environmental variables on lycopene’s vibrational characteristics, particularly regarding electronic occupancy, molecular orbitals, and the detailed exploration of vibrational and electronic states. This constraint impedes a comprehensive understanding of the structure and activity relationships within complex biologically relevant systems. For instance, acquiring reliable Raman intensity data presents a substantial challenge for researchers, especially in the context of in situ experiments and biological specimens. The recorded signals frequently suffer from distortions caused by phenomena such as reabsorption and light scattering within the sample, resulting in low reproducibility of spectral intensity measurements. As a result, these constraints significantly hinder the accuracy and reliability of quantitative analyses that rely on Raman intensity data [12,13]. Interference from fluorescence in Raman spectroscopy and the influence of water in infrared absorption spectroscopy, particularly when analyzing biological samples, represent significant sources of experimental interference that cannot be overlooked [14,15]. These experimental limitations have greatly hindered the precise characterization of electronic placeholders, molecular orbital configurations, and complex vibrational interactions. Therefore, relying solely on experimental methods is insufficient to thoroughly understand the structure-activity relationship in complex systems; a comprehensive theoretical framework is needed.

To overcome these experimental hurdles, first-principles density functional theory calculations offer a powerful avenue to elucidate the environmental effects on lycopene at the molecular level [16]. A pivotal geometric parameter in this context is the bond length alternation (BLA), defined as the average difference in length between adjacent C-C single bonds and C=C double bonds along the polyene backbone [17]. The physical significance of BLA lies in its direct quantification of π-electron delocalization: a smaller BLA indicates a more equalized bond length distribution, reflecting extended electron delocalization and enhanced molecular stability [18]. This parameter serves as a sensitive structural probe that bridges molecular geometry with electronic properties. Specifically, the BLA diminishes, resulting in a reduced disparity between bond lengths. The C=C experiences elongation, whereas the C–C undergoes contraction. The molecule attains a state of bond length equilibrium, leading to a more thermodynamically stable configuration. The spatial extent of π-electron delocalization expands, thereby increasing the effective conjugation length [19]. Therefore, a reduction in BLA is accompanied by a narrowing of the HOMO–LUMO gap, as the delocalized π-electrons raise the HOMO energy and lower the LUMO energy, thereby reducing the vertical excitation energy and causing a characteristic red shift in the absorption spectrum.

Over the past two decades, computational studies have progressively elucidated the structural, electronic, and vibrational properties of carotenoids. Chasse et al. [20] established the relative stability of lycopene isomers at the HF/3-21G level. Methodological benchmarks by Requena et al. [21] and Liu et al. [22] validated the B3LYP functional for carotenoid geometry and vibrational spectra, while Macernis et al. [23] demonstrated its robustness for substituted derivatives. Novikov et al. [24,25] systematically correlated Raman spectra with conjugation length and end/side group structures across multiple carotenoids. More recently, conceptual DFT analyses [26] and studies on carotenoid–cyclodextrin complexes [27] have extended these insights to reactivity and environmental interactions. Building on this foundation, the present work focuses specifically on the solvent-induced modulation of lycopene’s electronic and vibrational properties, a dimension that remains underexplored despite its relevance to biological environments.

To address this fundamental issue, the present study utilizes first-principles computational simulations to systematically examine the influence of environmental modulation on the electronic states and vibrational dynamics of lycopene. Particular emphasis is placed on charge redistribution, orbital energetics, and specific vibrational characteristics. Specifically, the lycopene molecule was modeled and subjected to density functional theory calculations to obtain characteristic information such as bond lengths, vibrational modes, and simulated Raman spectra. Analyzed and compared the structural configurations, vibrational patterns, charge distributions, orbital energies, electronic energy levels, and oscillator strengths of the lycopene molecule under different polarity environments. By providing molecular-level insights into these interactions, this work not only elucidates the properties of lycopene’s photophysical behavior but also establishes a predictive framework for optimizing its stability and bioavailability, thereby enhancing its potential applications in nutrition, healthcare, and pharmaceutical formulations.

## 2. Results and Discussion

### 2.1. Geometric Structure and Electronic Properties

**Figure 1:**
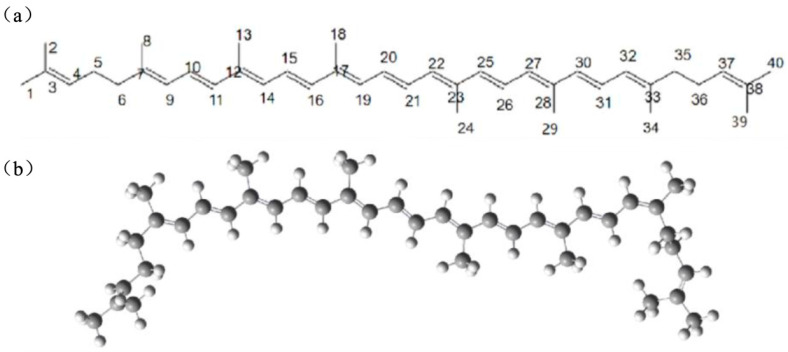
(**a**) Molecular structure schematic and (**b**) model diagram of lycopene. The numbers represent the carbon atom numbers. The black balls and gray balls represent carbon atoms and hydrogen atoms, respectively.

To investigate the impact of local environmental polarity on lycopene, molecular models were developed with the molecule situated within various solvent media. The solvents chosen for this analysis comprised water and methanol, representing highly polar environments; benzaldehyde and acetophenone, representing weakly polar solvents; and 1,1,1-trichloroethane and cyclohexane, representing non-polar solvents. This deliberate variation in solvent polarity facilitates a thorough examination of how solvation influences the electronic and structural characteristics of lycopene. Figure 1a presents a schematic representation of lycopene. Figure 1b illustrates the molecular structure of lycopene using a ball-and-stick model, where gray spheres denote carbon atoms and white spheres represent hydrogen atoms. Structurally, lycopene is characterized by an extended conjugated double bond system forming a polyene framework. The carbon backbone consists of alternating single and double bonds, creating a highly conjugated, linear, and acyclic arrangement. The molecule is functionalized with ten methyl groups and exhibits a symmetric configuration.

Geometric analysis indicated that the carbon backbone of lycopene demonstrates significant extensibility, characterized by a pronounced alternation in bond lengths between shorter double bonds and longer single bonds. This pattern of bond length alternation contributes to a certain degree of structural rigidity and enhances planarity throughout the conjugated chain. The conjugated framework facilitated extensive electron delocalization throughout the polyene chain. This electron delocalization imparts lycopene with significant optical characteristics, enabling it to absorb light within the visible spectrum and manifest a red coloration, which constitutes the structural basis for its function as a natural pigment. Additionally, the extended conjugation contributes to increased molecular stability and imparts unique chemical reactivity; lycopene is capable of interacting with reactive entities such as free radicals, thereby underpinning its strong antioxidant activity. Furthermore, the arrangement of terminal groups and methyl substituents along the carbon chain affected both steric hindrance and the distribution of electron density [28]. The resulting spatially extended conjugated system underpins lycopene’s capacity to participate in diverse intermolecular interactions.

**Figure 2:**
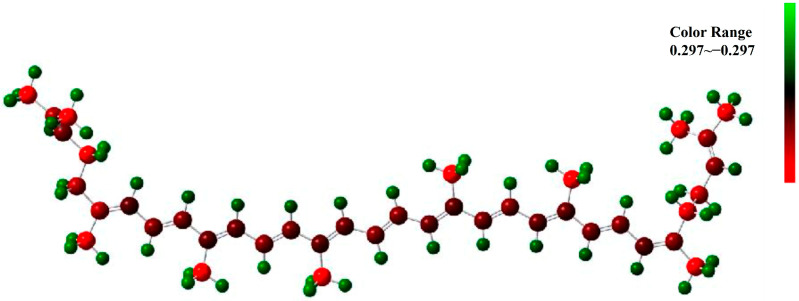
Electrostatic potential map of lycopene. Green and red indicate positive and negative electrostatic potential, respectively. Darker shades correspond to smaller absolute values, and lighter shades to larger values.

The charge distribution of lycopene exhibits a regular pattern, as illustrated in Figure 2. Within the electrostatic potential map, green hues correspond to atoms bearing partial positive charges, whereas red hues denote atoms with partial negative charges. The intensity of these colors reflects the magnitude of the respective charges, as indicated by the accompanying color scale. The linear conjugated double bond system characteristic of lycopene results in a distinctive charge distribution along its carbon framework. Carbon atoms exhibit negative partial charges. Those located in the central region of the carbon backbone display a less intense coloration relative to terminal and methyl-substituted carbons, suggesting a comparatively weaker negative charge in the central region. Carbon atoms at the molecular termini and within methyl groups possess more pronounced negative charges. Additionally, variations are observed among hydrogen atoms; although all hydrogens carry partial positive charges, those bonded to methyl groups exhibit lighter coloration, indicative of a relatively stronger positive charge compared to hydrogens directly attached to the main carbon chain.

To quantify the charge distribution along the carbon backbone of lycopene and further investigate the influence of polar environments on its charge populations, partial atomic charges were computed under various solvent conditions, including water, methanol, benzaldehyde, acetophenone, 1,1,1-trichloroethane and cyclohexane. The resulting charge values for carbon atoms under different environments were systematically summarized in Table 1. Regarding the total charge on carbon atoms, the highest values were recorded in water and methanol, with water exhibiting a total carbon charge of −7.02 and methanol showing −6.939. Benzaldehyde and acetophenone, which represent weakly polar solvents, displayed slightly lower charge values of −6.881 and −6.885, respectively. A further reduction was observed in 1,1,1-trichloroethane, with a value of −6.726. The lowest absolute charge magnitude was found in cyclohexane, with a value of −6.242. This trend clearly demonstrates a correlation between increasing solvent polarity and enhanced electron redistribution in lycopene. The solvents can be ranked in descending order of polarity as follows: water induced the strongest polarization, followed by methanol, benzaldehyde, acetophenone, 1,1,1-trichloroethane, and finally cyclohexane, which exhibited the weakest effect. The variation in the absolute charge values exhibits a consistent trend with solvent polarity, further supporting the correlation between enhanced electronic polarization and increasing polar solvation.

Based on the numbering convention established in Figure 1a, carbon atoms C1, C2, C39, and C40 demonstrate elevated absolute charge values, all of which are situated within the terminal methyl groups. Similarly, carbon atoms C8, C13, C18, C24, C29, and C34 exhibit relatively high absolute charge magnitudes; these correspond to methyl groups attached to the central conjugated polyene chain. Among these, the charges associated with C13, C18, C24, and C29 remain notably stable and are largely unaffected by variations in the solvent environment. Atoms C9, C12, C17, C23, C28, and C32 present lower charge values. It is noteworthy that the absolute charge values of C19 and C22 differ markedly between aqueous and methanolic solvents. The charge distributions observed in benzaldehyde and acetophenone are strikingly similar. Atoms C1, C2, C8, C13, C18, C24, C29, C39, and C40 exhibit significant variability across different solvents, with fluctuations reaching approximately –0.3, indicating a pronounced sensitivity to solvent effects. In contrast, the charge values of C9, C12, C17, C23, and C28 display minimal variation across solvents, with maximum differences near –0.1, suggesting a lower sensitivity to solvation. Additionally, the overall charge distribution along the carbon framework preserves symmetry consistent with the molecular architecture.

### 2.2. Molecular Orbitals

The redistribution of charge indicates that solvents with varying polarity induce alterations in the electronic environment of lycopene, changes that are closely associated with the energy levels and composition of its molecular orbitals. The molecular orbital characteristics of lycopene were calculated to further analyze the effects of solvent interactions on the molecular orbital properties and electron cloud distribution of lycopene.

**Figure 3:**
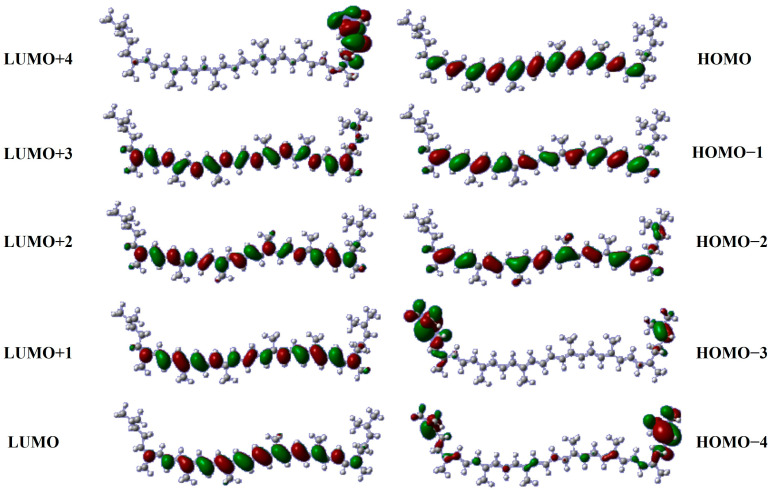
Isosurface plots of molecular orbitals of lycopene in a cyclohexane environment. The color of the equipotential surfaces represents the difference in orbital phase.

The electronic transition from the highest occupied molecular orbital (HOMO) to the lowest unoccupied molecular orbital (LUMO) defines the first excited state in the majority of molecules and is fundamentally associated with their chemical stability. In this study, cyclohexane, characterized as a weakly polar solvent, serves as the medium for the analysis depicted in Figure 3, which presents the frontier molecular orbitals of lycopene alongside additional nearby orbitals of significance. The spatial distribution of electron density within the pertinent molecular orbitals reveals distinct and characteristic patterns. In the LUMO+4 orbital, electron density is primarily localized on the terminal methyl group situated on the right side. The LUMO+3 orbital exhibits electron density concentrated on two hydrogen atoms of the left moiety, with minor contributions from three carbon atoms on the right, while the remaining density is distributed along the central carbon chain. For the LUMO+2 orbital, electron density is observed on two hydrogen atoms on the left and one carbon atom on the right, with the principal distribution centered along the central carbon framework. A notable shift in localization occurs in the LUMO+1 orbital, where electron density is found on one hydrogen atom on the left, as well as on one carbon and one hydrogen atom on the right. In contrast, both the LUMO and HOMOs demonstrate complete delocalization of electron density throughout the entire central polyene chain. The HOMO−1 orbital presents minor electron density on two hydrogen atoms on the left side and significant density on one hydrogen and one carbon atom on the right, with the majority of the density residing along the central chain, where it exhibits a propensity toward destabilization. Considerable electron density in the HOMO−2 orbital is observed on two hydrogen atoms on the left, two carbon atoms, and one hydrogen atom on the right, with a pronounced concentration along the central chain and minor contributions from the associated hydrogen atoms. The HOMO−3 orbital is characterized by a high electron density localized on the left methyl group, accompanied by moderate density on the right methyl group. Lastly, the HOMO−4 orbital displays electron density heavily concentrated on the right methyl group, with moderate density on the left methyl group and minimal density along the central carbon chain.

The electronic excitation characteristics of the lycopene molecule are predominantly influenced by the delocalization properties of its conjugated main chain. Analysis of the frontier molecular orbitals and adjacent orbitals reveals that the charge density is primarily localized on the carbon framework, signifying that electronic transitions within the conjugated backbone govern the low-lying excited-state electronic transitions of lycopene. Specifically, the highest occupied molecular orbital (HOMO) electron density is chiefly distributed over the carbon–carbon double bonds, whereas the lowest unoccupied molecular orbital (LUMO) electron density is mainly associated with the carbon–carbon single bonds. This distribution implies that the transition from the ground state to the first excited state in lycopene is predominantly characterized by π–π* transitions along the conjugated carbon chain. As the orbital energy levels deviate from the frontier region, the electron cloud progressively shifts towards the termini, concurrently developing an asymmetric distribution profile. This observed asymmetry indicates that terminal groups participate more actively in electronic transitions of higher energy levels.

Importantly, the orbital energy distribution and the corresponding charge localization are intimately linked to the BLA parameter, which quantifies the degree of π-electron delocalization along the polyene backbone. A smaller BLA value reflects enhanced electron delocalization, which in turn narrows the HOMO–LUMO gap and facilitates the π–π* transition. This geometric–electronic correlation is consistent with the seminal computational work of Chasse et al., who investigated the structural and electronic properties of seven lycopene isomers [20]. They reported HOMO and LUMO energies for each isomer, providing early insights into how geometric isomerization modulates the electronic structure of lycopene. This combined analysis provides a unified framework for rationalizing the solvent-dependent Raman spectral shifts observed experimentally.

When the solvent environment is varied, only minimal changes in the electron density distribution are detected. The molecular orbital diagrams for the other five solvent environments are illustrated in Figures S1–S5, and their associated orbital energies are compiled in Table 2. In the table, the leftmost column indicates the orbital sequence indices, with orbitals 149 and 148 corresponding to the LUMO and HOMO, respectively, and the remaining orbitals numbered sequentially thereafter. As solvent polarity decreases, a general decline in molecular orbital energies is observed. The reduction in energy is relatively modest in the first four solvent environments, approximating 0.01 eV. However, more substantial energetic deviations emerge in 1,1,1-trichloroethane and cyclohexane, where the orbital energy lowering reaches approximately 0.06 eV compared to the strongly polar aqueous environment. The energy gap between the frontier molecular orbitals is typically larger than that observed between molecular orbitals located further from the frontier. This suggests that the frontier molecular orbitals exhibit a heightened sensitivity to variations in the solvent environment. This pattern suggests that polar solvents preferentially stabilize the HOMO, corresponding to the electron-dense C=C bonds within the polyene backbone, by means of strengthened electrostatic interactions with the solvent’s dielectric environment. The concomitant narrowing of the HOMO–LUMO gap directly translates to a red shift in the S_0_→S_2_ vertical excitation energy. Importantly, this solvent-induced gap reduction correlates inversely with the BLA parameter; a smaller BLA reflects greater π-electron delocalization, which raises the HOMO energy and lowers the LUMO energy, thereby reducing the gap. This geometric–electronic coupling provides a unified mechanistic framework for understanding how environmental perturbations modulate lycopene’s optical properties.

**Figure 4:**
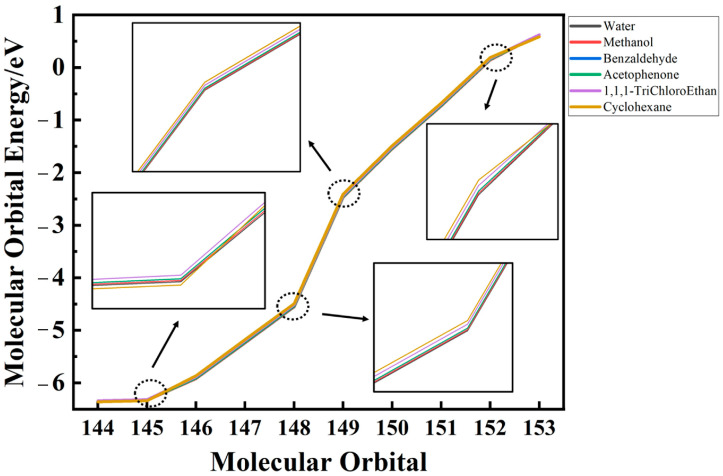
The molecular orbital energy diagram of lycopene in six different solvents. The horizontal axis represents the orbital index (from HOMO−5 to LUMO+5), and the vertical axis represents the orbital energy (in eV). The inset panels magnify selected regions of the energy diagram.

To effectively illustrate the variations in the energy levels of molecular orbitals, a line graph as depicted in Figure 4 was constructed. Between the HOMO−3 and HOMO−2 orbitals, the energy curve for cyclohexane exhibits a steeper increase compared to the other five solvents. Conversely, between the LUMO+3 and LUMO+4 orbitals, its trajectory is surpassed by the others. This pronounced variability indicates that cyclohexane experiences the most significant energetic perturbations among the six solvents in response to changes in orbital energy levels. Meanwhile, the curve for 1,1,1-trichloroethane remains substantially distanced from the others, suggesting that the orbital energetics of lycopene are considerably influenced within this specific solvent environment. The polar solvents, water and methanol, exhibit the lowest orbital energies, corresponding to the bottom two curves in the figure. This depression in energy may be attributed to the potential role of hydrogen bonding in these solvents, which could enhance the stability of the π-system. In contrast, cyclohexane, likely due to its inability to engage in polar interactions, displays the highest orbital energies, represented by the uppermost curve. The weakly polar nature of 1,1,1-trichloroethane results in an energy profile akin to that of non-polar solvents, though its orbital energy is slightly lower than that of cyclohexane. The curves for benzaldehyde and acetophenone are situated between those of the strong and weak polar solvents. Collectively, these observations support a general inverse correlation between solvent polarity and molecular orbital energy: higher polarity correlates with lower orbital energies, while lower polarity is associated with elevated orbital energies.

### 2.3. Raman Spectroscopy and Vibration Modes

**Figure 5:**
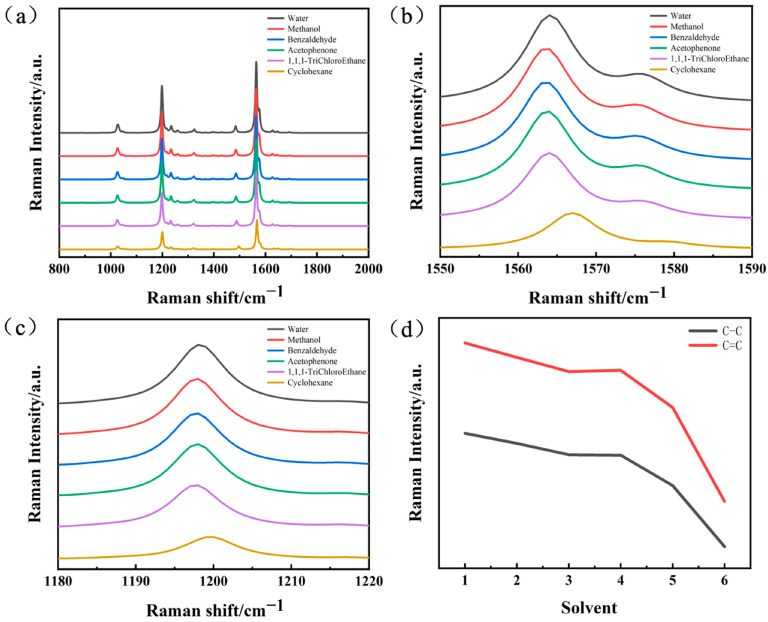
Raman Spectra of Lycopene. (**a**) The spectral range from 800 to 2000 cm^−1^ encompasses all principal vibrational modes. (**b**) The region of 1550 and 1590 cm^−1^, corresponding to the peak of carbon-carbon double bonds. (**c**) The region of 1180 to 1220 cm^−1^, corresponding to the peak of carbon-carbon single bonds. (**d**) A comparative analysis of the intensities of the carbon-carbon vibrational peaks, with labels 1 through 6 indicating water, methanol, benzaldehyde, acetophenone, 1,1,1-trichloroethane, and cyclohexane, respectively.

The linear conjugated system of lycopene demonstrates significant sensitivity to environmental influences. Various solvent matrices can alter the molecular vibrational states through specific interactions, such as hydrogen bonding and dipole–dipole interactions, resulting in discernible changes in Raman spectra, including shifts in peak positions and variations in intensity. Figure 5 illustrates the Raman spectra of lycopene in different solvent environments. Distinct characteristic peaks are observed near 1200 cm^−1^ and 1600 cm^−1^, which correspond to the stretching vibrations of carbon−carbon single bonds and carbon-carbon double bonds within the linear conjugated framework, respectively. Additionally, minor satellite peaks adjacent to these main features arise from carbon-hydrogen bending vibrations, while Raman signals related to C-H stretching vibrations are almost imperceptible.

To validate the reliability of our computational protocol, we compared the calculated Raman shifts of lycopene with experimental values reported in the literature [27]. The results validated that the B3LYP/6-311+G(d,p) level of theory offers an accurate representation of the vibrational characteristics of lycopene, thereby supporting its application in the systematic investigation of solvent effects conducted in this study.

The Raman spectral profiles display negligible shifts in peak positions across various solvent environments, with cyclohexane showing the most pronounced deviation of approximately 3 cm^−1^ relative to other solvents. In contrast, significant variations are evident in peak intensities. Within a given solvent environment, the characteristic peak intensity associated with carbon-carbon double bonds surpasses that of carbon-carbon single bonds, indicative of differences in the scattering cross-sections of the respective vibrational modes. Across different solvents, aqueous solutions present the highest characteristic peak intensities, followed by a clear decreasing trend corresponding to reductions in solvent polarity. The influence of the solvent on spectral characteristics primarily arises from intermolecular interactions that modify the distribution of electron density and alter vibrational energy barriers. Highly polar solvents interact extensively with the polar functional groups of lycopene, leading to a redistribution of electron density and consequent changes in the force constants associated with carbon-carbon bonds. These modifications are expected to result in slight shifts in peak positions.

Raman spectroscopy serves as an effective tool for elucidating the characteristics of chemical bonds, wherein the bond strength is fundamentally determined by the bond order of the molecular orbitals constituting the bond. Chemical bonds with higher bond orders exhibit larger force constants, which correspond to elevated vibrational frequencies. Conversely, bonds with lower bond orders possess smaller force constants, resulting in reduced vibrational frequencies. In the case of conjugated polyenes such as lycopene, the HOMO is characterized as a bonding π orbital, with electron density predominantly localized within the C=C regions. Given that the HOMO functions as a bonding π orbital along the entire conjugated chain, it confers a significantly high bond order to the C=C bonds, thereby causing the C=C stretching vibrational mode to manifest in the high-frequency region near 1500 cm^−1^. In contrast, the LUMO is identified as a weak π antibonding orbital, associated with a relatively low bond order for the C-C. This lower bond order results in the C-C stretching vibrational mode appearing in the lower frequency region around 1100 cm^−1^. Meanwhile, this trend is a direct spectroscopic manifestation of sole-induced BLA reduction. As the BLA decreases, the π-electron delocalization along the polyene backbone is enhanced, which weakens the C=C bond force constant and consequently lowers the ν_1_ vibrational frequency. This interpretation is consistent with the established structure–Raman correlations for carotenoids: Novikov et al. [24,25] demonstrated that an increase in conjugation length (which corresponds to a smaller BLA) induces a monotonic red-shift of the C=C stretching band across different carotenoid molecules.

Raman activity is directly proportional to the square of the derivative of the polarizability tensor with respect to the vibrational coordinate. A pronounced Raman signal associated with a particular vibrational mode indicates that this mode effectively induces changes in the molecular polarizability. The static polarizability of a molecule can be represented as the cumulative contribution of all occupied molecular orbitals, with particular emphasis on the HOMO, due to its elevated energy level and susceptibility to perturbation. The C=C stretching vibration exhibits the highest Raman activity because it substantially modifies the length of the conjugated π-electron system and the extent of electron delocalization, thereby exerting a dominant influence on the molecular polarizability governed primarily by π molecular orbitals.

In contrast to the minimal variations observed in Raman shift, Raman activity exhibits a pronounced decreasing trend with the reduction in solvent polarity. As solvent polarity diminished, the extent of electron density redistribution within lycopene’s π-system became progressively attenuated. This reduced perturbation results in more modest alterations to the molecular polarizability tensor, which is the fundamental determinant of Raman scattering intensity. In these less polar environments, the diminished stabilization of excited electronic states leads to a decrease in the derivative of polarizability with respect to normal coordinates, consequently reducing the Raman cross sections for key vibrational modes.

## 3. Computational Details

The calculation was performed using the DFT method in the Gaussian 09 software package [29]. The optimization was computed via analytic energy second derivatives under the B3LYP/6-311+G(d,p) level [30,31]. Frequency calculations were subsequently performed at the same level to confirm the absence of imaginary frequencies and to obtain the harmonic vibrational frequencies. Raman activities were derived from the frequency calculation outputs. These activities were then converted into relative Raman intensities using the following relationship:

(1) $$I_{i}\propto \frac{{\left(\nu_{0}-\nu_{i}\right)}^{4}}{\nu_{i}}S_{i}$$

where ν_0_ is the frequency of the incident radiation, ν_i_ is the vibrational frequency of the ith normal mode, and S_i_ is the corresponding Raman scattering activity. Raman spectra were fitted using Lorentz line shapes. All the calculations were carried out by selecting a single molecule, and the SMD solvent model was selected to simulate the solution environment [32]. As an implicit solvent approach, the SMD model was widely utilized in computational studies. Rather than explicitly representing the detailed structure and spatial distribution of solvent molecules surrounding the solute, SMD treats the solvent environment as a polarizable continuum. This model simultaneously accounts for both the polar and non-polar components of the solvent medium. GaussView 5.0 was used for wave function analysis, including molecular visualization, vibration mode analysis, spectral data acquisition, molecular orbital simulation, etc. Atomic partial charges were computed using Mulliken population analysis based on the wavefunctions optimized in each solvent environment.

It should be noted that the B3LYP functional, in its standard form, does not explicitly include long-range dispersion corrections. While such corrections are important for intermolecular interactions and conformational preferences of large conjugated systems, the primary focus of the present work is on the intramolecular properties of lycopene under different solvation environments, with particular emphasis on solvent-induced trends rather than absolute energetics. For such intramolecular properties, B3LYP has been extensively validated for carotenoid systems in the literature [21,22,23,33,34]. Nevertheless, we acknowledge this as a limitation of the present approach, and future investigations employing dispersion-corrected functionals would be valuable to further refine the conformational and energetic analyses. The choice of the 6-311+G(d,p) basis set represents a pragmatic balance between computational cost and accuracy.

All calculations were performed on the all-trans isomer of lycopene, which is the predominant and most thermodynamically stable form in natural systems and serves as the sample in most experimental Raman investigations. For each solvent environment, the initial molecular structure was constructed in the all-trans configuration and was then subjected to full, unconstrained geometry optimization without imposing any symmetry constraints or freezing any internal coordinates. During these independent optimizations, all geometric parameters were allowed to fully relax until the maximum force and displacement convergence criteria were satisfied. This protocol guarantees that the optimized geometries, along with the corresponding electronic and vibrational properties determined for each solvent, inherently reflect the solvent-induced geometric relaxation at the local energy minimum. It should be noted, however, that a systematic global conformational search is beyond the scope of the present work, and our conclusions are drawn specifically for the all-trans conformation under the given solvation models.

The text was translated and the language was polished using AI tools. The AI tool used is DeepSeek-V3.

## 4. Conclusions

Based on the comprehensive investigation presented, the photophysical properties of lycopene are demonstrably governed by its extensive conjugated polyene framework and exhibit marked sensitivity to solvent microenvironment. Geometric and electronic analyses reveal a delocalized π-system characterized by significant bond length alternation, which underpins the molecule’s pronounced optical absorption in the visible region and its potent antioxidant capacity. Solvent-induced polarization effects manifest systematically in the electronic structure: with increasing solvent polarity, enhanced electron density redistribution along the carbon backbone is observed, accompanied by a general stabilization of molecular orbital energies. Frontier molecular orbital analysis revealed that the HOMO–LUMO transition, which is primarily characterized as a π–π* excitation localized along the conjugated backbone, constitutes the principal factor governing the low-lying excited state.

Raman spectroscopic analysis indicates that peak positions remain largely consistent across different solvents, with the exception of cyclohexane, which causes a slight shift. In contrast, peak intensities demonstrate significant variability depending on the solvent. Specifically, aqueous environments produce the highest Raman activity, with a noticeable decrease in intensity observed as solvent polarity diminishes. This pattern is attributed to a reduction in the polarizability derivative within non-polar media. These findings elucidate a coherent structure–property–solvation relationship, confirming that solvent polarity not only affects the electronic distribution and orbital energetics of lycopene but also directly impacts its vibrational response. Consequently, this provides a robust framework for predicting the behavior of lycopene across a range of dielectric environments.

## Figures and Tables

**Table 1:** Carbon atom charge of lycopene in different solvent environments (Unit: e).

	Water	Methanol	Benzaldehyde	Acetophenone	1,1,1-TriChloroEthane	Cyclohexane
C1	−0.282	−0.281	−0.28	−0.28	−0.276	−0.266
C2	−0.284	−0.284	−0.282	−0.282	−0.279	−0.268
C3	−0.154	−0.153	−0.152	−0.152	−0.15	−0.143
C4	−0.144	−0.142	−0.14	−0.14	−0.133	−0.111
C5	−0.231	−0.231	−0.231	−0.231	−0.232	−0.233
C6	−0.166	−0.165	−0.165	−0.165	−0.163	−0.159
C7	−0.157	−0.157	−0.157	−0.157	−0.157	−0.156
C8	−0.278	−0.277	−0.276	−0.276	−0.273	−0.262
C9	−0.109	−0.107	−0.105	−0.104	−0.096	−0.073
C10	−0.148	−0.138	−0.136	−0.136	−0.13	−0.111
C11	−0.132	−0.13	−0.129	−0.128	−0.123	−0.107
C12	−0.102	−0.1	−0.099	−0.099	−0.094	−0.083
C13	−0.295	−0.295	−0.295	−0.295	−0.294	−0.289
C14	−0.115	−0.113	−0.111	−0.111	−0.105	−0.086
C15	−0.142	−0.14	−0.138	−0.138	−0.132	−0.115
C16	−0.123	−0.122	−0.121	−0.121	−0.116	−0.101
C17	−0.099	−0.098	−0.096	−0.096	−0.091	−0.079
C18	−0.295	−0.295	−0.295	−0.295	−0.294	−0.289
C19	−0.114	−0.113	−0.111	−0.111	−0.106	−0.089
C20	−0.137	−0.135	−0.133	−0.133	−0.127	−0.109
C21	−0.137	−0.135	−0.133	−0.133	−0.127	−0.109
C22	−0.144	−0.113	−0.111	−0.111	−0.106	−0.089
C23	−0.099	−0.098	−0.096	−0.096	−0.091	−0.08
C24	−0.295	−0.295	−0.295	−0.295	−0.294	−0.289
C25	−0.123	−0.122	−0.121	−0.12	−0.116	−0.101
C26	−0.142	−0.14	−0.138	−0.138	−0.132	−0.115
C27	−0.114	−0.112	−0.11	−0.11	−0.103	−0.085
C28	−0.102	−0.101	−0.099	−0.099	−0.095	−0.084
C29	−0.297	−0.297	−0.297	−0.297	−0.295	−0.29
C30	−0.131	−0.129	−0.127	−0.127	−0.122	−0.105
C31	−0.133	−0.132	−0.13	−0.13	−0.125	−0.109
C32	−0.117	−0.115	−0.113	−0.113	−0.105	−0.081
C33	−0.129	−0.129	−0.128	−0.128	−0.126	−0.123
C34	−0.265	−0.264	−0.263	−0.263	−0.26	−0.251
C35	−0.225	−0.225	−0.225	−0.225	−0.225	−0.222
C36	−0.189	−0.189	−0.189	−0.189	−0.188	−0.186
C37	−0.153	−0.151	−0.148	−0.148	−0.14	−0.118
C38	−0.151	−0.15	−0.15	−0.15	−0.148	−0.141
C39	−0.281	−0.281	−0.28	−0.279	−0.276	−0.265
C40	−0.286	−0.285	−0.284	−0.284	−0.281	−0.27

**Table 2:** The molecular orbital energy of lycopene (eV).

	Water	Methanol	Benzaldehyde	Acetophenone	1,1,1-TriChloroEthan	Cyclohexane
153	0.62506	0.62533	0.62506	0.62506	0.62261	0.58643
152	0.14334	0.14878	0.15531	0.15613	0.17435	0.1923
151	−0.73168	−0.7257	−0.71862	−0.71781	−0.69795	−0.67755
150	−1.54768	−1.54115	−1.53326	−1.53245	−1.51042	−1.4843
149	−2.46786	−2.4616	−2.45371	−2.4529	−2.43086	−2.40856
148	−4.55165	−4.54539	−4.5375	−4.53669	−4.51438	−4.49344
147	−5.23845	−5.23219	−5.22403	−5.22322	−5.2001	−5.17616
146	−5.92035	−5.91437	−5.90648	−5.90594	−5.88472	−5.86568
145	−6.33189	−6.32917	−6.3259	−6.32563	−6.31829	−6.33869
144	−6.35256	−6.35038	−6.34794	−6.34766	−6.34168	−6.36018

## Data Availability

The data that support the findings of this study are available from the corresponding author upon reasonable request.

## Supplementary Materials

The following supporting information can be downloaded at: https://www.mdpi.com/article/10.3390/molecules31132358/s1, Figure S1: Molecular orbital diagram of lycopene in water; Figure S2: Molecular orbital diagram of lycopene in methanol; Figure S3: Molecular orbital diagram of lycopene in benzaldehyde; Figure S4: Molecular orbital diagram of lycopene in acetophenone; Figure S5: Molecular orbital diagram of lycopene in 1,1,1-trichloroethane.
