# Supplementary material for: Environmental Factors Modulate the Electronic Transitions and Molecular Vibrations of Lycopene: A Spectroscopy Perspective
Source: Molecules. 2026 Jul 3;31(13):2358. doi: 10.3390/molecules31132358 (PMC13363628; doi:10.3390/molecules31132358)
Supplement: Supplementary file 1 [file molecules-31-02358-s001.zip › molecules-4356652-supplementary.pdf]

Supporting materials:

Environmental Factors Modulate the Electronic Transitions and Molecular Vibrations of Lycopene: A Spectroscopy Perspective

Lu Xing,[a*] *Shuping Zhao,*[a] *Yeqiu Li,*[a] *Yi Shi,*[a] *Qin Dai,*[a] *Wei Zhang,* [b]

a. School of Science, Shenyang Ligong University, NO.6 Nanping Central Road

Shenyang 110159, China

b. School of Chemistry and Chemical Engineering, Nanjing University of Science and

Technology. Nanjing 210094, China

[*]The Corresponding Author: xinglu@sylu.edu.cn (L. Xing)

Frontline molecular orbital diagrams of lycopene in other environments：

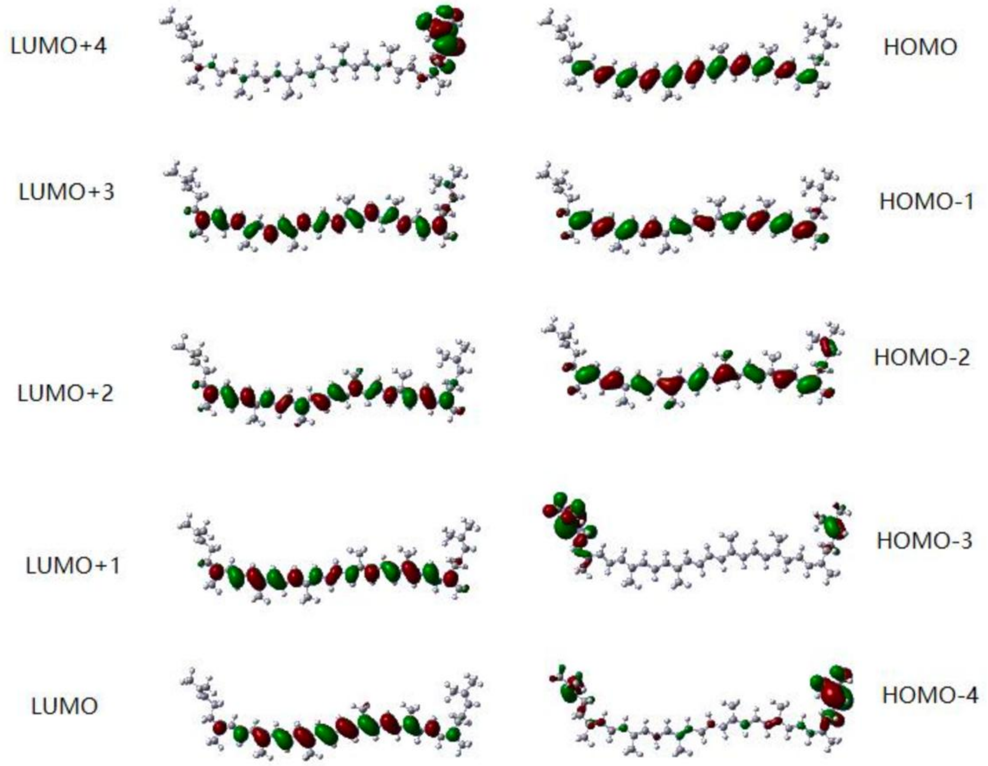

Fig. S1 Molecular orbital diagram of lycopene in water

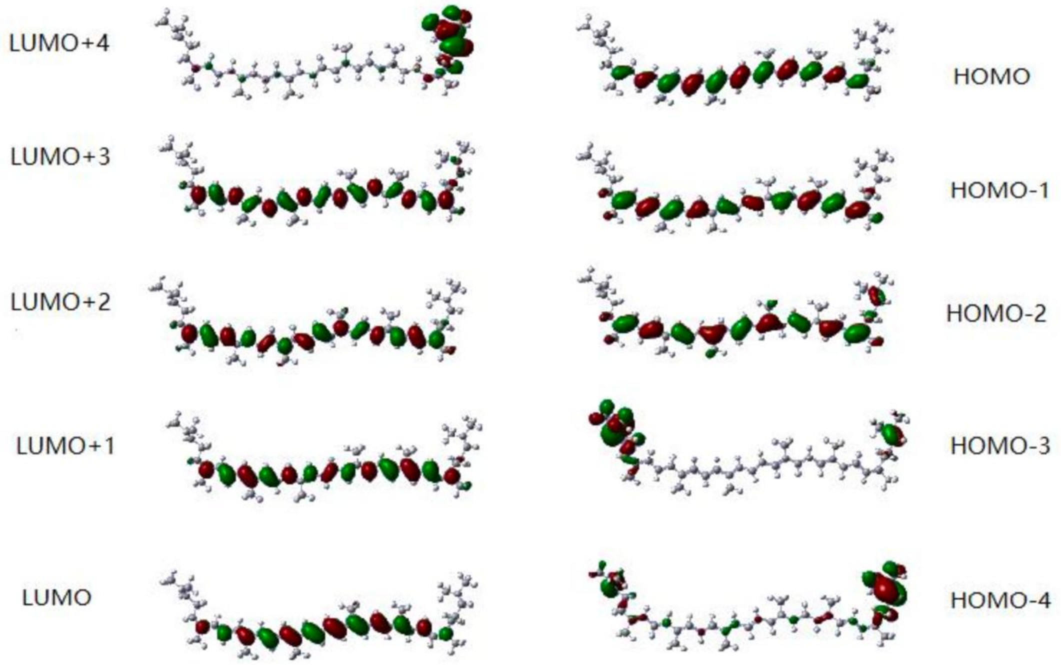

Fig. S2 Molecular orbital diagram of lycopene in methanol

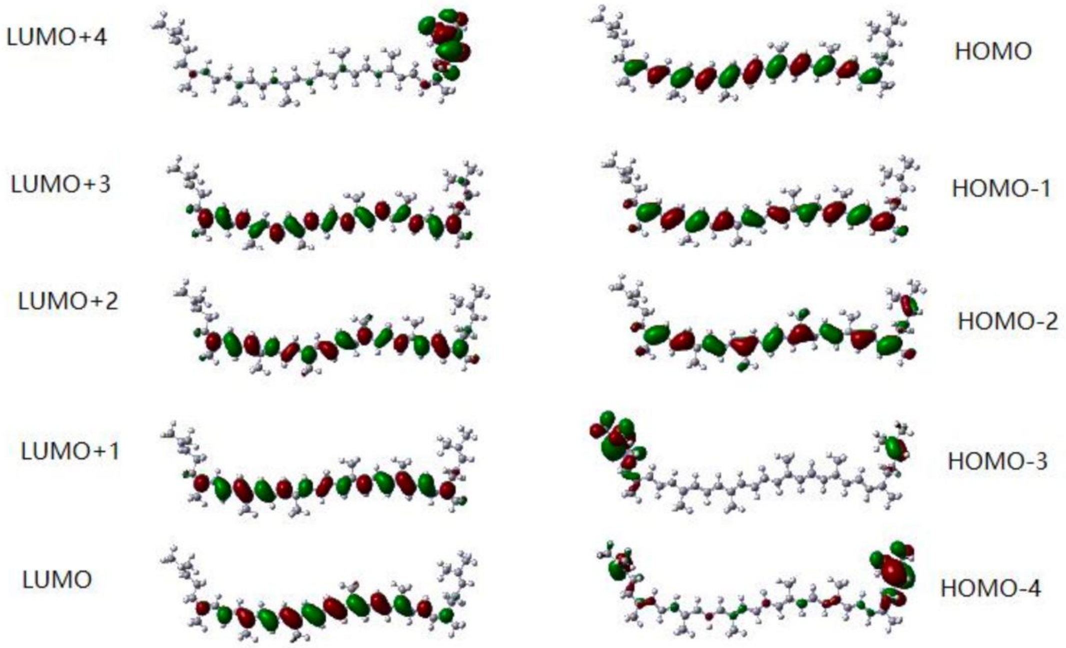

Fig. S3 Molecular orbital diagram of lycopene in benzaldehyde

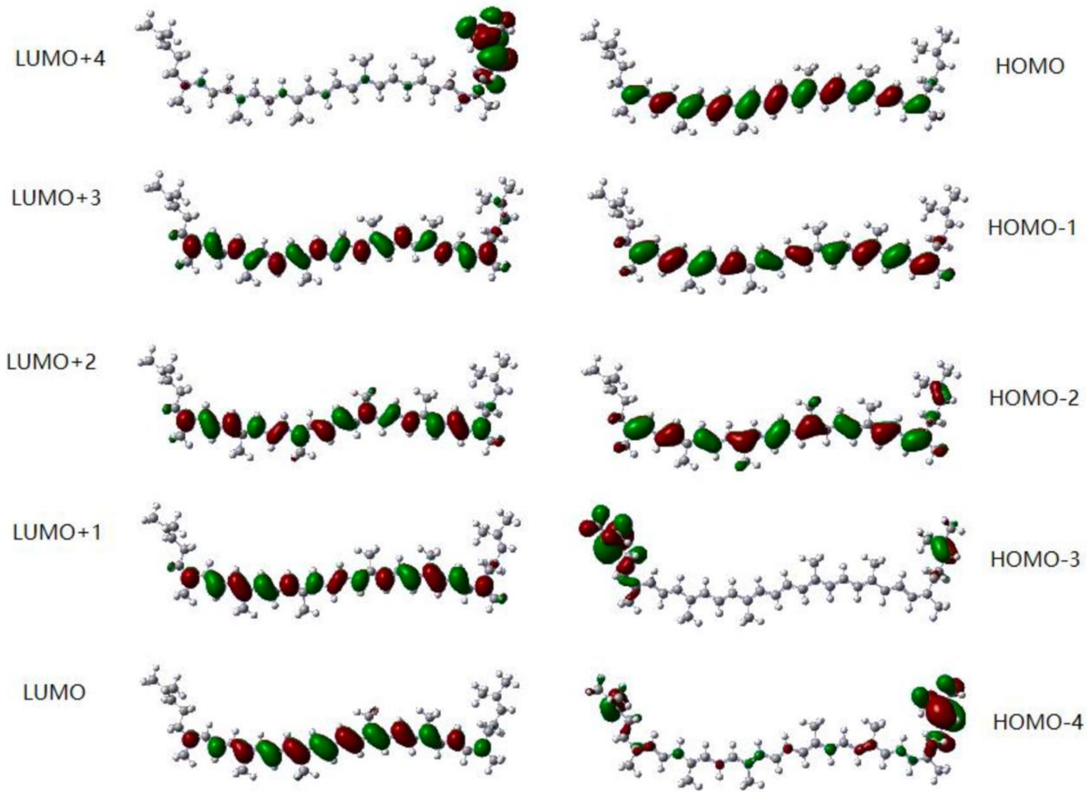

Fig. S4 Molecular orbital diagram of lycopene in acetophenone

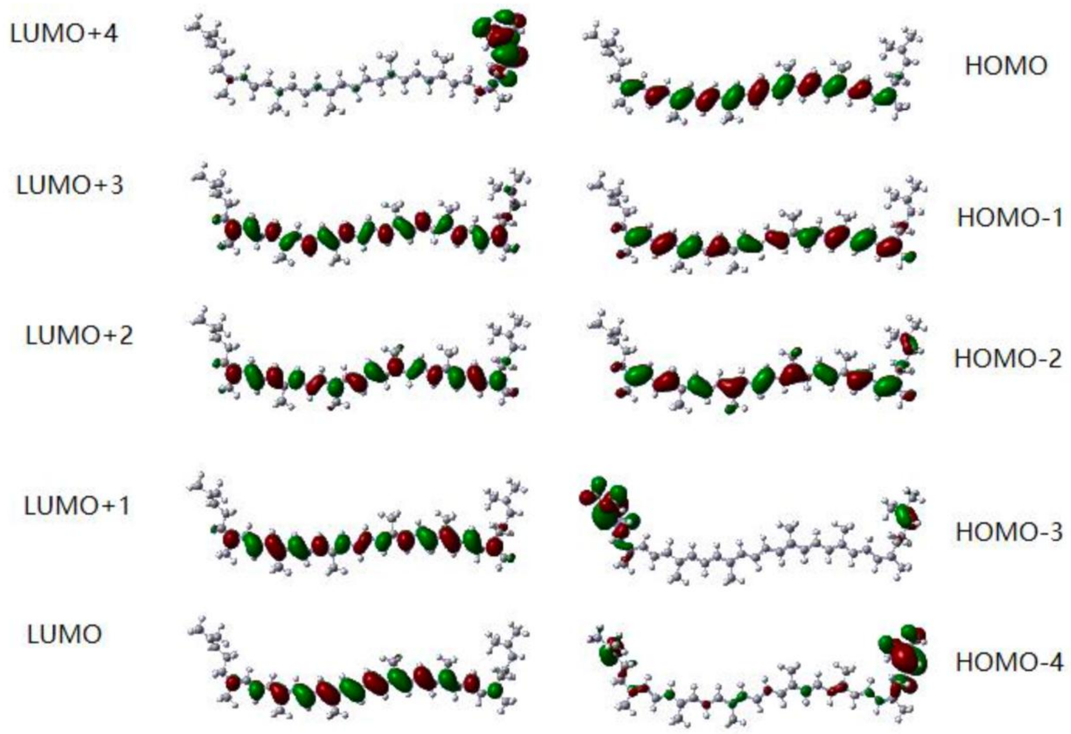

Fig. S5 Molecular orbital diagram of lycopene in 1,1,1-trichloroethane
